# *Trypanosoma cruzi* Cell Death Induced by the Morita-Baylis-Hillman Adduct 3-Hydroxy-2-Methylene-3-(4-Nitrophenylpropanenitrile)

**DOI:** 10.1371/journal.pone.0093936

**Published:** 2014-04-08

**Authors:** Jana M. Sandes, Adriana Fontes, Carlos G. Regis-da-Silva, Maria C. A. Brelaz. de Castro, Claudio G. Lima-Junior, Fábio P. L. Silva, Mário L. A. A. Vasconcellos, Regina C. B. Q. Figueiredo

**Affiliations:** 1 Departamento de Microbiologia, Centro de Pesquisas Aggeu Magalhães, Recife, PE, Brazil; 2 Departamento de Biofísica e Radiobiologia, Universidade Federal de Pernambuco, Recife, PE, Brazil; 3 Laboratorio de Biologia Parasitaria, Centro Pesquisas Gonçalo Moniz, Salvador, BA, Brazil; 4 Departamento de Imunologia, Centro de Pesquisas Aggeu Magalhães, Recife, PE, Brazil; 5 LASOM-PB, Departamento de Química, Universidade Federal da Paraíba, João Pessoa, PB, Brazil; Albert Einstein College of Medicine, United States of America

## Abstract

Chagas disease, caused by the protozoan *Trypanosoma cruzi*, remains a serious health concern due to the lack of effective vaccines or satisfactory treatment. In the search for new compounds against this neglected disease, we have previously demonstrated that the compound 3-Hydroxy-2-methylene-3-(4-nitrophenylpropanenitrile) (MBHA3), derived from the Morita-Baylis-Hillman reaction, effectively caused a loss of viability in both the epimastigote and trypomastigote forms. However, the mechanisms of parasite death elicited by MBHA3 remain unknown. The aim of this study was to better understand the morphophysiological changes and the mechanism of cell death induced by MBHA3 treatment on *T. cruzi*. To perform this analysis, we used confocal microscopy and flow cytometry to monitor the fluorescent probes such as annexin-V/propidium iodide (AV/PI), calcein-AM/ethidium homodimer (CA/EH), acridine orange (AO) and rhodamine 123 (Rho 123). Lower concentrations of MBHA3 led to alterations in the mitochondrial membrane potential and AO labeling, but did not decrease the viability of the epimastiogote forms, as determined by the CA/EH and AV/PI assays. Conversely, treatment with higher concentrations of MBHA3 led to extensive plasma membrane damage, loss of mitochondrion membrane potential, DNA fragmentation and acidification of the cytoplasm. Our findings suggest that at higher concentrations, MBHA3 induces *T. cruzi* epimastigote death by necrosis in a mitochondrion-dependent manner.

## Introduction

*Trypanosoma cruzi*, the etiological agent of Chagas disease, is one of the most serious infectious pathogens to humans, with 10 million people infected worldwide, mostly in Latin America, and 100 million people at risk of acquiring Chagas disease. Despite their high toxicity, the drugs nifurtimox and benznidazole are the only available treatment for this illness. Although these drugs are effective against acute infections, their efficacy in the chronic phase of the disease remains controversial, and no consensus on the evaluation of a parasitological cure has been achieved [1]. In this regard, the development of more effective, low cost drugs, without significant side effects is still needed for the treatment of Chagas disease.

**Figure 1:**
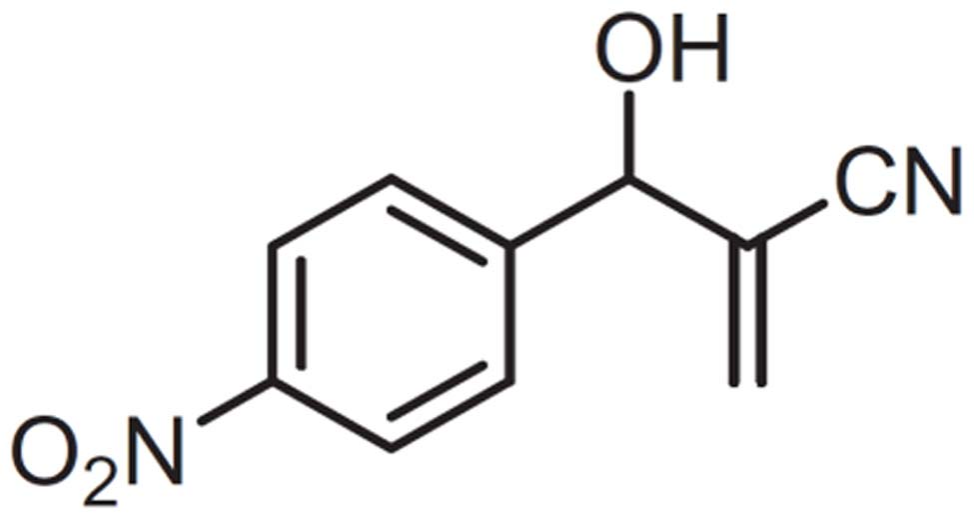
Chemical structure of MBHA3.

In a search for new compounds against Chagas disease, we have previously demonstrated that the incubation of parasites with the Morita-Baylis-Hillman adduct, 3-Hydroxy-2-methylene-3-(4-nitrophenylpropanenitrile, MBHA3 (Figure 1), had profound effects on the growth of epimastigotes forms and caused a loss of trypomastigote viability, with IC_50_/LC_50_ for epi- and trypomastigotes of 28.5 and 25.5 μM respectively. Ultrastructural analysis of *T. cruzi* treated with MBHA3 revealed morphological characteristics of programmed cell death (PCD) [2].

PCD is a genetically regulated active process that plays a central role in the development and homeostasis of multicellular organisms and is associated with a wide variety of human diseases, including immunological and developmental disorders, neurodegeneration and cancer. Cell death involves three major mechanisms: apoptosis, autophagy and necrosis [3]. Apoptosis is an orchestrated process that occurs in both normal and pathological conditions. The morphological hallmarks of apoptosis include chromatin condensation and nuclear fragmentation, which are usually followed by a rounding up of cells. Finally, apoptotic cells give rise to small round bodies that are surrounded by a membrane and contain intact organelles and nuclear fragments [4]. In addition to the morphological changes, three main biochemical events can be observed in cells undergo apoptosis 1) caspase activation, 2) DNA and protein breakdown and 3) phosphatidylserine exposition in the outer layer of plasma membrane [5]. Autophagy is a physiological mechanism that involves the sequestration of excess, old and unneeded cytoplasmic organelles and long-lived macromolecules, into large double-membrane vesicles, called autophagosomes, followed by subsequent delivery of the cargo into lysosomes for degradation, with no inflammatory response [6]–[8]. Necrosis is usually defined as a process of cell collapse that involves an increase in cell volume (oncosis), which ultimately leads to plasma membrane rupture and the unorganized dismantling of swollen organelles [9]. Apart from the presence of plasma membrane permeabilization, necrosis lacks specific biochemical markers.

Essential features of PCD, such as the genes encoding the basic cell death machinery and their morphological and biochemical characteristics, appear to be conserved in nematodes, [10]–[11], insects [12]–[13] and vertebrates (mammals) [14]. As in multicellular organisms, it has been demonstrated that various stimuli, such as drugs, oxidative stress, starvation, exposure to human serum, inhibition of signaling molecules, etc., are able to elicit a PCD-like response in an increasing number of unicellular eukaryotic species [15], including kinetoplastid parasites of the genus *Leishmania* [16]–[17] and *Trypanosoma* [2], [18]. Although electron microscopy has proven to be useful for the identification of drug target organelles and the determination of PCD phenotypes [19], in the case of MBHA3-induced cell death, this tool was not sufficiently robust to unequivocally discriminate between different phenotypes of PCD [2]. Therefore, we used the combination of confocal microscopy, flow cytometry, and fluorescent probes to examine the mechanisms involved in *T. cruzi* epimastigote death induced by MBHA3. Understanding the mechanism involved in the drug-induced cell death of this parasite may provide insights into the pathogenesis of Chagas disease and help to better develop therapies against this illness.

## Materials and Methods

### Drug treatment

The MBHA3 compound (Figure 1) was synthesized and characterized as previously described [20]. The MBHA3 was initially dissolved in dimethyl sulfoxide (DMSO) at a concentration of 250 mM. This solution was diluted into culture medium to obtain a stock solution at 5 mM (stock solution). The stock solution was diluted again in the culture medium to obtain concentrations of 28.5, 57.0 and 114.0 μM, which correspond to the previously determined values of IC_50_, 2x IC_50_ and 4x IC_50_ after 72 hours of treatment respectively [2]. Throughout the experimental procedures the concentration of DMSO never exceeded 0.05%, which is non-toxic to the protozoa.

### Parasites

All experiments were carried out using *T. cruzi* epimastigote forms (Dm28c) from axenic cultures, maintained in liver infusion tryptose (LIT) medium supplemented with 10% fetal bovine serum (FBS) at 28°C and harvested during the exponential phase of growth.

### Annexin V and propidium iodide labeling

Parasites, that were non-treated or treated with different concentrations of MBHA3 for 24, 48 and 72 hours, were incubated with an Annexin V-FITC Apoptosis Detection Kit (Sigma-Aldrich, St Louis, USA) following the manufacturer's instructions. Briefly, after drug treatment, the parasites were harvested by centrifugation, washed with PBS and incubated for 15 minutes at 28°C with 5 μg/mL annexin-V-FITC (AV) and 10 μg/mL propidium iodide (PI) diluted in annexin-V binding buffer. Next, the cells were centrifuged, resuspended in phosphate buffered saline (PBS) and immediately analyzed on a laser confocal scanning microscope or flow cytometer. For confocal microscopy, both probes were excited with a 488 nm diode laser and the fluorescence emission was recorded at 510 and 560 nm for AV and PI respectively. The samples were observed under a Leica SPII/AOBS (Mannheim, Germany) scanning confocal microscope. Dual-parameter flow cytometric analysis was performed with the flow cytometer FACS-Calibur (Becton-Dickinson, San Jose, CA, USA), using a 530/30 nm signal detector (FL1-H) for AV-FITC and a 582/42 nm PI emission signal detector (FL2-H). The fluorescence intensity was acquired for 20,000 events, and the data were analyzed using Cell-Quest™ (Becton–Dickinson, San Jose) and expressed as the percentage of cells in each population phenotype (unstained, stained only with PI, stained only with AV or stained with both markers) compared to the total number of cells analyzed.

### Live/Dead assay

Cell viability was assessed by a LIVE/DEAD Viability/Cytotoxicity Kit (Molecular Probes, Eugene, Oregon, USA). Control (non treated) and MBHA3-treated parasites were harvested by centrifugation after 72 hours of incubation and resuspended in 0.5 mL PBS containing 0.1 μM of calcein and 8 μM of ethidium homodimer. Samples were incubated for 30 minutes at room temperature and immediately analyzed by flow cytometry with a 530/30 nm filter (FL1-H) for calcein and a 670 nm long pass filter (FL3-H) for the ethidium homodimer. For confocal microcopy, treated and control cells were incubated in PBS with 4 μM of calcein and 4 μM of ethidium homodimer. Cells were observed using a 488 nm laser and, images were acquired and analyzed using the same parameters described for the AV/PI labeled-cells.

### Rhodamine 123 (Rho) and acridine orange (AO) assays

Treated and non-treated parasites were washed and resuspended in 0.5 mL PBS with 10 μg/mL Rho 123 (Sigma-Aldrich, St Louis, USA) or 10 μg/mL AO (Sigma-Aldrich, St Louis, USA) for 20 minutes. After loading, the parasites were washed in PBS and immediately analyzed by flow cytometry, and the fluorescence intensities for AO (acid compartments) and Rho 123 (mitochondrial membrane potential) were quantified. A total of 20,000 events were acquired in the region previously established as corresponding to *T. cruzi* epimastigotes, based on the forward (FSC) and side (SSC) scatter. Alterations in the fluorescence intensities of Rho 123 (FL1-H) or AO (FL3-H) were quantified by the index of variation (IV) that was obtained by the equation (TM − CM)/CM, where TM is the median of fluorescence for treated parasites and CM is that of the control (non-treated). AO-labeled parasites were also observed by a confocal microscope using 488 and 543 nm lasers.

### DNA fragmentation

After 72 hours of incubation with MBHA3, treated and untreated parasites were harvested by centrifugation, washed in PBS and assayed for DNA fragmentation as previously described [21]. Briefly, the cells pellets (10^7^ epimastigotes) were lysed with sarkosyl detergent lysis buffer (50 mM Tris, 10 mM EDTA, 0.5% w/v sodium-N-lauryl sarcosine, pH 7.5) and the supernatant digested with proteinase K (20 mg/mL) for 2 hours at 50°C. The sample was then treated with RNase A (0.3 mg/mL) for 1 hour at 37°C. The lysates were then extracted with phenol/chloroform (25∶24), precipitated in cold ethanol and subjected to electrophoresis on 1% agarose gels containing ethidium bromide. DNA fragments were visualized under UV light.

## Results

### Effects of MBHA3 on the viability of *T. cruzi*

**Figure 2:**
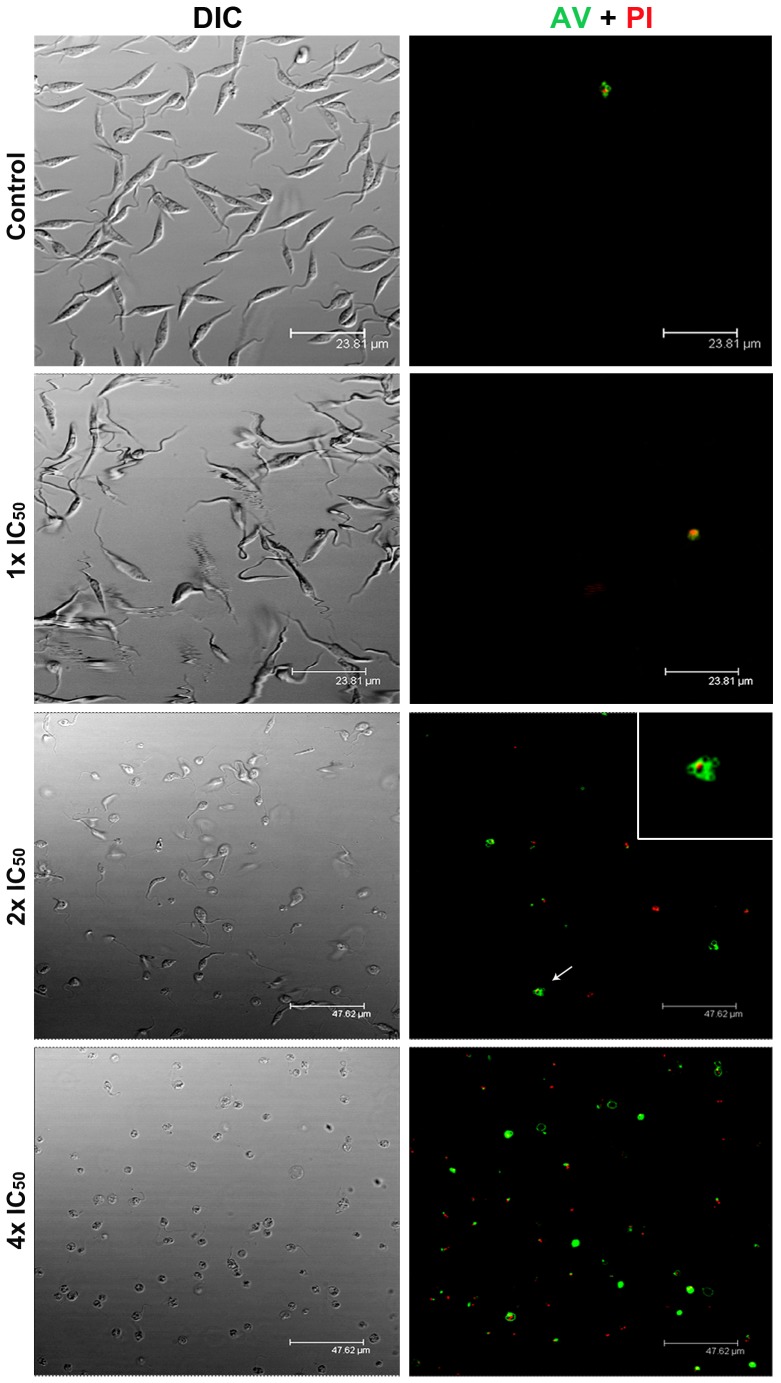
Effects of BMHA3 treatment on Anexin V/PI labeling. Confocal microcopy analysis of control and MBHA3-treated parasites labeled with AV/PI, after 72 hours of incubation. Note the presence of AV and PI double positive cells presenting membrane blebs at the 2x IC_50_ of MBHA3 (arrow and inset). Drastic morphological changes could be observed by differential interference contrast (DIC, left column) at the 2x and the 4x IC_50_ of MBHA3.

Annexin V is a Ca^2+^-dependent phospholipid-binding protein with a high affinity for phosphatidylserine, whereas PI is a cell-impermeant fluorescent dye that intercalates DNA and RNA of cells whose plasma membrane integrity has been lost. By using these fluorescent markers, our flow cytometry analysis showed increased cell death in parasites treated with MBHA3, with major differences detected in cells treated with the 4x IC_50_/72 h. Regardless of the incubation time, at lower concentrations of the drugs (1x and 2x IC_50_), the percentage of non-apoptotic/intact cells (AV^−^/PI^−^) was always greater than 90%. Late apoptotic (AV^+^/PI^+^) and necrotic phenotypes (AV^−^/PI^+^) were preponderant in cells treated with 4x IC_50_/72 h, corresponding to approximately 63% of cell population, while 8.84% of the epimastigote population was AV^+^/PI^−^, and 28.7% of cells were negative for both markers (AV^−^/PI^−^) (Table 1). Because the effects of MBHA3 were more noticeable at 72 hours, we used this incubation time for all further analysis. Confocal microscopy assays of cells labeled with AV/PI corroborated the data obtained by flow cytometry. Incubation of epimastigotes with 28.5 μM of MBHA3 (1x IC_50_) (Figure 2) did not yield a substantial labeling for either markers compared to the control, for which most of the cells were unstained (AV^−^/PI^−^) after treatment. Conversely, at drug concentrations corresponding to the 2x and the 4x IC_50_ values, an increase of AV^+^/PI^−^, AV^+^/PI^+^ and AV^−^/PI^+^ phenotypes, which are indicative of intense cell death, could be observed. Morphological changes associated with apoptosis, such as nuclear material trapped in plasma membrane blebs, were also observed in AV^+^/PI^+^ positive cells treated with the 2x IC_50_ (Figure 2, inset). A large number of cells undergoing necrosis cell death without the translocation of phosphatidylserine (AV^−^/PI^+^) were mostly detected at the 4x IC_50_.

**Table 1:** Flow cytometric analysis of *T. cruzi* epimastigotes treated with MBHA3 and labeled with Annexin V/propidium iodide.

	% cells											
AV/PI	Control			1x IC_50_			2x IC_50_			4xIC_50_		
	24 h	48 h	72 h	24 h	48 h	72 h	24 h	48 h	72 h	24 h	48 h	72 h
AV^−^/PI^−^	99.18	98.87	98.75	98.7	97.26	95.2	98.77	95.07	90.61	57.66	57.88	28.7
AV^+^/PI^−^	0.54	0.66	0.85	0.68	1.93	3.21	0.59	1.94	3.42	1.13	2.43	8.84
AV^+^/PI^+^	0.17	0.33	0.31	0.46	0.58	1.25	0.36	2.24	4.87	31.95	37.39	42.18
AV^−^/PI^+^	0.11	0.14	0.09	0.16	0.23	0.34	0.28	0.75	1.10	9.76	12.30	20.28

*The values represent the mean of two independent experiments.

**Figure 3:**
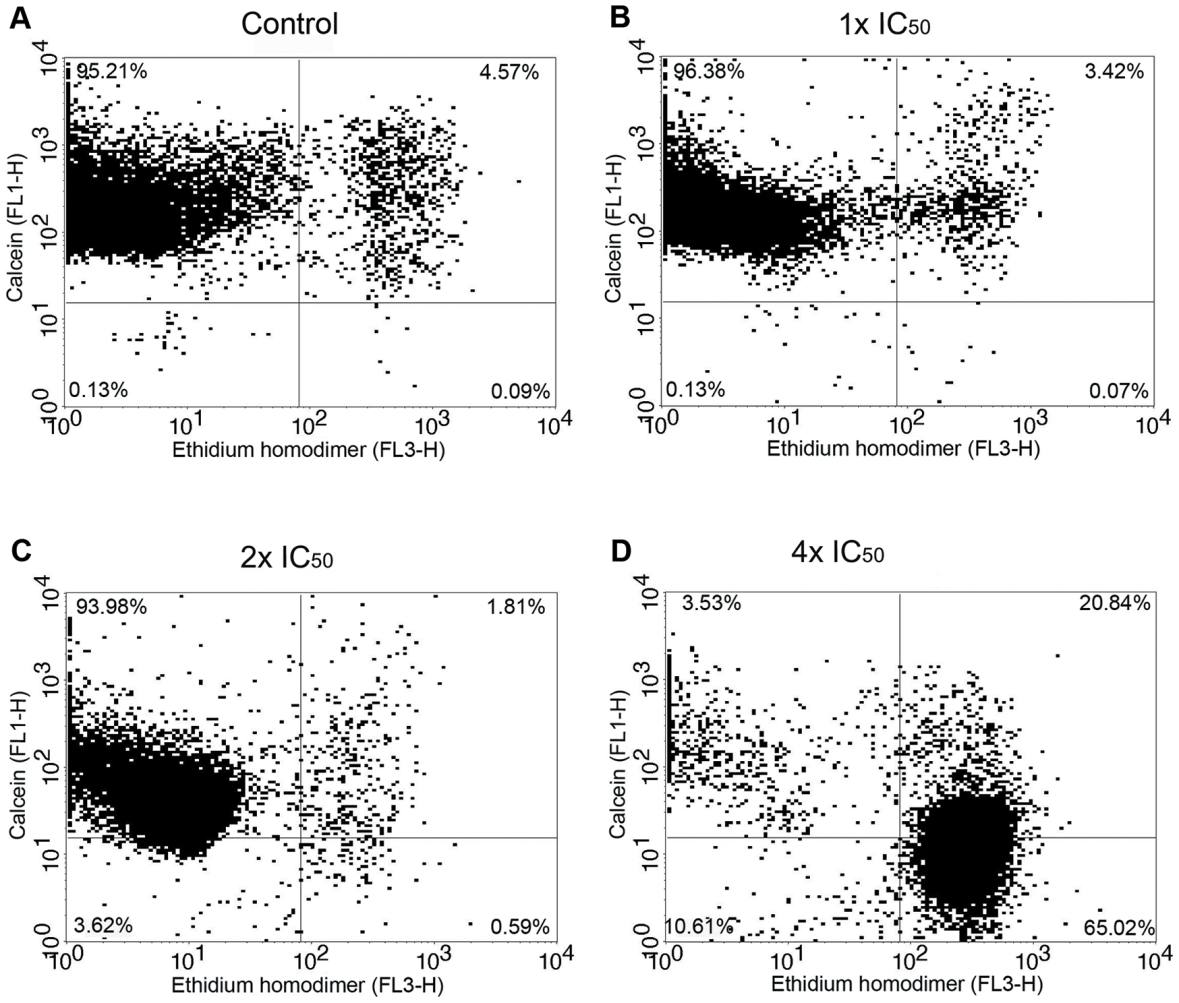
Cytometry analysis of the effects of MBHA3 on Calcein AM/EH. Flow cytometric dot plots of control (A) and MBHA3-treated cells (B – D) labelled with CA/EH. Cells in the upper left quadrant were positive only for calcein. Cells in the lower right quadrant were positive only for ethidium heterodimer. Events in the upper right quadrant were double positive for both markers, whereas the lower left quadrant corresponds to the cells negative for the both dyes. The dot plots are representative of duplicate experiments with similar results. There were 20,000 recorded events/experimental condition.

In an attempt to clarify whether non-apoptotic/intact parasites presenting an AV^−^/PI^−^ phenotype, undoubtedly corresponded to viable cells, the Calcein-AM (CA) and ethidium homodimer-1 (EH) live/dead viability test was performed. Flow cytometric analysis showed a dose-dependent decrease of CA^+^/EH^−^ cells, followed by a corresponding increase in CA^+^/EH^+^ and CA^−^/EH^+^ cells (Figure 3). It is important to note that even in those cells stained only with CA, a considerable decrease in the fluorescence intensity for this marker could be observed at the 2x IC_50_ of MBHA3 (Figure 3C). In all concentrations, the double-negative cells (CA^−^/EH^−^) did not reach rates higher than 10% of the total of cell population.

**Figure 4:**
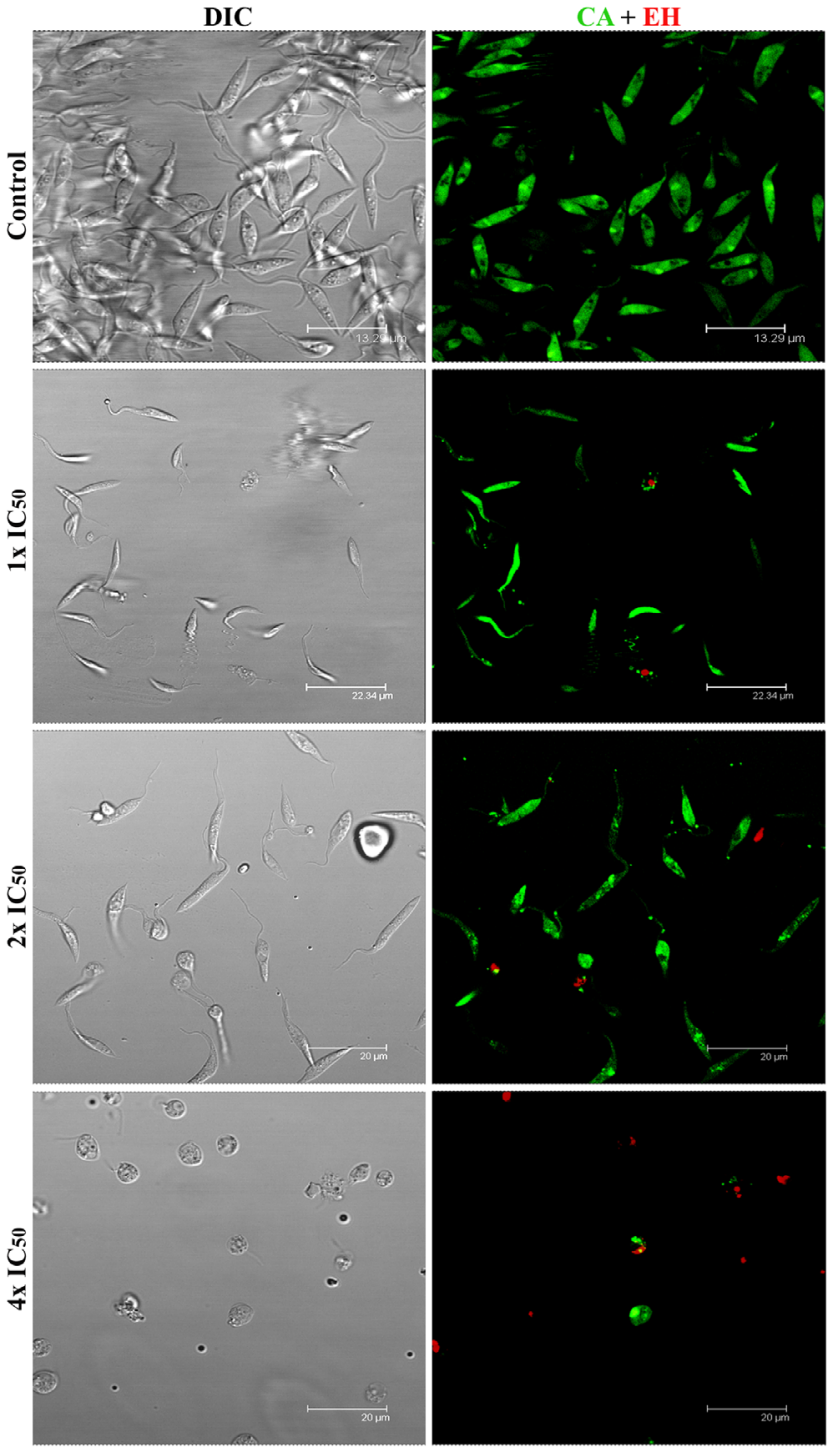
Confocal microscopy of control and MBHA3-treated parasites submitted to CA/EH labelling. Control cells presenting normal morphology as observed by DIC (left column) and CA bright green fluorescence indicative of intense esterase activity. Few control cells were labeled with EH (red). However, a dose-dependent loss of green fluorescence with corresponding increase in red fluorescence could be observed in the treated cells. Note in the 4x IC_50_ treated-cells the presence of swollen parasites.

The confocal images showed that most untreated cells exhibited homogeneous, bright calcein green fluorescence in the cytoplasm, nucleus and kinetoplast, while few cells were stained red (CA^+^/EH^−^) (Figure 4). No considerable difference in this profile could be detected in cells incubated with IC_50_ of MBHA3. However, in cells treated at the 2x IC_50_ of MBHA3, the labeling pattern of CA was affected, and a punctuated and heterogeneous labeling became evident, indicating a gradual loss of esterase activity. A considerable decrease in the CA^+^/EH^−^ population and increase in the CA^+^/EH^+^ population could be observed in cells treated with the 4x IC_50_.

**Figure 5:**
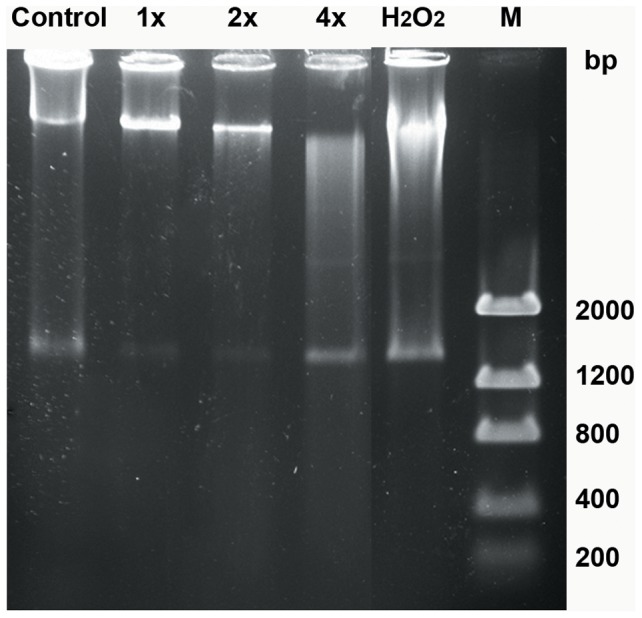
DNA fragmentation induced by MBHA3 on *T. cruzi* epimastigote forms. Electrophoresis of total DNA of the control and MBHA3-treated epimastigote forms. Non-specific DNA fragmentation could be observed in cells treated with the 2x IC_50_ and the 4x IC_50_ MBHA3. H_2_O_2_ was used as a positive control of parasite death. M = Low DNA Mass Ladder.

Consistent with the findings above, the treatment of cells with the 2x IC_50_ and the 4x IC_50_ of MBHA3 or 4 mM of H_2_O_2_ (used as a positive control for inducing cells death) led to an increased DNA smearing, a characteristic of autolytic DNA breakdown, compared to the IC_50_-treated cells and the control (Figure 5).

### Effects of MBHA3 on the acidic compartments of *T. cruzi*

**Figure 6:**
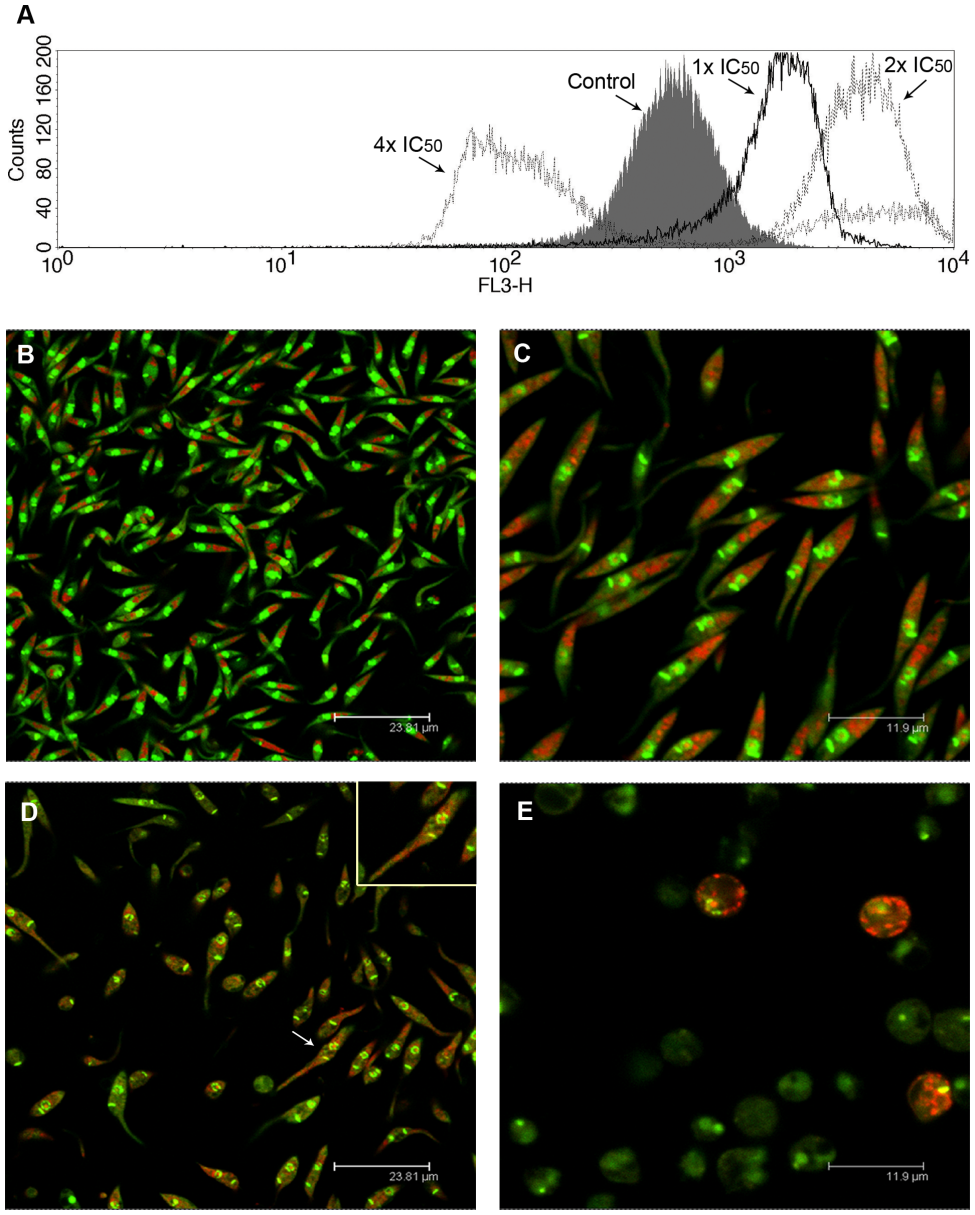
Effects of MBHA3 treatment on the acidic compartments of T. cruzi. (A) Overlay flow cytometric histograms of the control and treated-cells labeled with AO, after 72 hours of drug incubation. A gradual shift of the red fluorescence could be observed in the cells treated with the IC_50_ and the 2x IC_50_ of MBHA3. Nevertheless, in cells treated with the 4x IC_50_ of MBHA3, a striking decrease of the fluorescent signal from red channel was observed. (B–E). Confocal microscopy images of the control (B) and treated-cells (C–E). Control cells presented a normal morphology with a bright green nucleus, pale green cytoplasm and large red-labeled compartments at the posterior end of cells (B). Detail of the IC_50_-treated culture showing slight changes in the parasite nucleus. (C). Aspect of parasite culture treated with the 2x IC_50_ of MBHA3 showing severe acidification of the parasite cytoplasm (D). Note the presence of the pyknotic nucleus (white arrow, inset). Parasites treated with the 4x IC_50_ of MBHA3 showed a round-shape body and a decrease in both green and red AO fluorescent signals (E).

The treatment of epimastigotes with the IC_50_ and 2x IC_50_ of MBHA3 led to an increase in the AO red fluorescence intensity, as observed by flow cytometry (Figure 6A). This result was confirmed by positive IV values of +2.01 and +1.9 for the IC_50_ and the 2x IC_50_ MBHA3, respectively. However, at the higher drug concentration, a striking decrease of AO red fluorescence could be observed (IV = −0.9). To test whether the increase of red fluorescence at lower drug concentrations was due to an increase in the number of acidic compartments or to general cytoplasm acidification, control and treated cells were observed by confocal microscopy. Control cells stained with AO presented a well-preserved morphology with bright green labeling in the nucleus and kinetoplast and a pale green fluorescence in the cytoplasm (Figure 6B). Red fluorescence was detected mainly within large organelles located at the posterior end of the cells, which correspond to the reservosome, a pre-lysosomal compartment in epimastigote forms. Small red vesicles were observed randomly distributed throughout the cytoplasm. In the IC_50_-treated cells, we observed a discrete increase in the number of red-labeled compartments and a few cells already presenting nuclear alterations (Figure 6C). Treatment with the 2x IC_50_ of MBHA3 caused a decrease in the green fluorescence and acidification of the cytoplasm (Figure 6D). At this concentration, the effects of the drug on the parasite nucleus became more pronounced, with intense nuclear pyknosis and karyorrhexis (Figure 6D, inset). Dramatic morphological changes, including cell body swelling and a completely loss of red labeling could be observed in most of cells at the higher MBHA3 concentration (Figure 6E).

### Effects of MBHA3 on the mitochondrial membrane potential of *T. cruzi*

**Figure 7:**
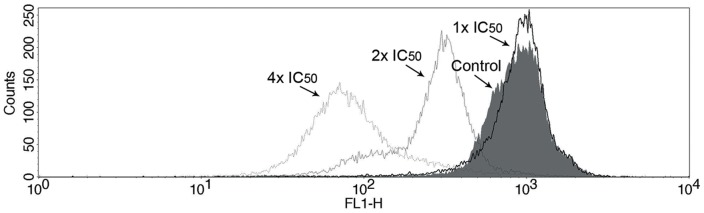
Effects of MBHA3 on the mitochondrial membrane potential. Overlay flow cytometric histograms of the control and the MBHA3-treated cells labeled with Rho 123 (FL1-H). The dose-dependent reduction of Rho 123 fluorescence intensity, mainly at the 4x IC_50_ of MBHA3, indicates the depolarization of the mitochondrial membrane. Histograms representative of duplicate experiments are shown.

To determine whether MBHA3-induced PCD of parasites was related to an alteration of the mitochondrial membrane potential (Δψm), we performed flow cytometry using Rho 123 staining. Incubation of the parasites with the IC_50_ of MBHA3 had no drastic impact on Rho 123 fluorescence intensity, as demonstrated by flow cytometry. The IV value at this concentration was −0.02. Conversely, at higher concentrations of MBHA3, gradual mitochondrion depolarization could be observed (Figure 7), with IV values of −0.64 and −0.87 at 2x IC_50_ and 4x IC_50_ of MBHA3, respectively.

## Discussion

Chagas disease, caused by the protozoa *Trypanosoma cruzi*, remains a serious health concern due a lack of effective vaccines and treatments. Efforts to control this endemic illness are based on therapeutic drugs, which have serious side effects and low efficacy during the chronic phase of disease. In addition, drug resistance has increased, and the cost of treatment is extremely expensive for most affected people. Thus, a search for new anti-parasitic agents for Chagas disease that have low patient toxicity and low cost is needed [22].

The Morita–Baylis–Hillman (MBH) reaction is a low cost, atom-efficient condensation method that provides easy access to highly functionalized carbonyl derivatives [20], [23]. MBH adducts are extensively used in the synthesis as versatile starting materials for many natural products and drugs. Previous work carried out by our group showed that at low concentrations, the compound 3-Hydroxy-2-methylene-3-(4-nitrophenylpropanenitrile), derived from the Morita-Baylis-Hillman reaction, effectively inhibited epimastigote growth and led to a decrease in trypomastigote viability. Ultrastructural analysis of treated-cells showed morphological characteristic that are associated with programmed cell death. However, the mechanisms of cell death elicited by MBHA3 treatment remain unknown [2].

In this work, we examined the effects of MBHA3 on the epimastigote form of *T. cruzi*. By using fluorescent markers, we attempted to better characterize the morphophysiological changes and the mechanism of cell death induced by MBHA3. Although this evolutive stage of the parasite is non-infective, epimastigotes are easily analyzed under confocal microscopy and flow cytometry; therefore, they are more suitable than trypomastigotes in physiological studies. Although there is experimental evidence that the mechanisms of cell death found in multicellular organisms are also present in unicellular organisms, including trypanosomatids, the evaluation of cell death in these parasites remains challenging [24]. For example, genes encoding caspases and death receptors, which are involved in cell death by apoptosis, are absent in trypanosomatids [25]. Thus, most studies on cell death in these microorganisms are still based on morphological parameters, mainly examined by transmission and scanning electron microscopy [26]. Although these techniques can provide valuable clues related to PCD in parasites, the ability to follow this process by direct live cell imaging is critical to better understanding the entire process [24], [27].

The evaluation of apoptosis and necrosis by both fluorescence microscopy and flow cytometry is usually accomplished by the combined use of annexin V-FITC, which accesses phosphatidylserine that is exposed on the external membrane in the early stage of apoptosis, and PI, which allows for the identification of nuclear alterations in the late stages of apoptosis or necrosis as a consequence of the increase in membrane permeability [9], [28]–[29]. Our results showed that the incubation of cells with MBHA3 at concentrations corresponding to once and twice the IC_50_ values did not yield significant losses in cell viability, although some morphological changes could be already identified. These results have been confirmed by other experiments, suggesting that MBHA3 has a more cytostatic than cytotoxic effect at the IC_50_ concentration. Most cells positive for AV were also PI positive, suggesting that apoptotic cells evolved into a secondary necrosis. However, we cannot rule out the possibility that annexin might also bind to the inner phosphatidylserine residues after the membrane integrity has been lost. It is usually assumed that annexin- and PI-negative cells (AV^−^/PI^−^) correspond to viable cells. However, this supposition should be considered carefully. It is possible that cell death mechanisms other than apoptosis or necrosis are operating in AV^−^/PI^−^ cells. To confirm this hypothesis, we used a CA/EH viability test to check whether the AV^−^/PI^−^ phenotype corresponds to live cells. According to the CA/EH viability test, we found that the viability was considerably lower than that reported from the AV/PI assay. The differences found between these methodologies were more pronounced in cell treated with 4x IC_50_ of the drug. Although both probes allowed for the detection of apoptotic and necrotic cells, annexin might be less effective than calcein in discriminating viable from dead cells. Thus, the use of the AV/PI assay alone could lead to an overestimate of live cells in culture [30].

AO is a nucleic acid-selective dye that emits green fluorescence upon DNA intercalation. AO also enters and becomes trapped in acidic vesicular organelles (AVOs), such as lysosomes [31]. In a low pH environment this dye emits red florescence. Thus, AO has been used, among other applications, to monitor major morphological changes induced by different stimuli and drug treatment [32]–[33]. We have found an increase in the red fluorescence intensity in cells treated with low concentrations of MBHA3. The increasing number of acidic compartments observed in the IC_50_-treated cells might be due to autophagy. Conversely, the diffuse red labeling observed throughout the cytoplasm in cells treated with 2x IC_50_ could be due to the deterioration of lysosomal membranes and acidification of the cytoplasm, leading to a loss of cell viability. The considerable decrease in green fluorescence found in cells treated with 4x IC_50_ MBHA3 could be due to damage or conformational alterations to nucleic acids, impairing AO-DNA intercalation. Consistent with this idea, we showed that MBHA3 induced a nonspecific DNA degradation at higher concentrations, resulting in a ‘smear’ of randomly degraded DNA, a feature commonly attributed to cell necrosis.

The mechanism of action by which MBHA3 induces cell death remains unsolved, but our previous molecular docking analyses have suggested that MBHA3 is a putative inhibitor of a *T. cruzi* farnesyl pyrophosphate synthase [2] a key enzyme in the mevalonate pathway in trypanosomes [34]–[36]. Furthermore, it has been proposed that the nitro groups present in MBHA3 might undergo a reduction leading to the oxidation of cellular constituents, such as nucleic acids and mitochondria, which are highly dependent on redox reactions [2], [37]–[38]. To test the latter hypothesis and investigate whether the MBHA3-induced apoptosis and necrosis of *T. cruzi* were related to alterations in the parasite mitochondrion we used Rho 123 to detect changes in the mitochondrial membrane potential. It has been shown that mitochondria play a pivotal role in cell death decisions [39]. In normal cells, the electrochemical gradient is maintained by active pumping of H^+^ during the transfer of electron throughout the respiratory chain. In this regard, the membrane potential maintains the integrity and function of mitochondria. Perturbations in this potential could lead to a decrease in ATP production and a reduction in the translation and transcription of mitochondrial genes, which ultimately results in apoptosis and/or necrosis [40]. Flow cytometry analysis of MBHA3-treated cells labeled with Rho 123 indicated a considerable loss of the mitochondrial membrane potential, even at low concentrations. Drug concentrations corresponding to IC_50_ and 2x IC_50_ led to a decrease of the Rho 123 fluorescence intensity, which cannot be attributed to plasma membrane permeabilization, because, at these concentrations no substantial labeling with PI could be detected. Our results showed that the alterations in mitochondria membrane potential, induced by MBHA3 treatment, preceded the *T. cruzi* cell death. As previously stated, the nitro groups of the MBHA3 molecules can induce the production of free radicals and reactive oxygen species, resulting in the decrease of mitochondrial membrane potential and cell death.

In conclusion our finds suggest that MBHA3, at higher concentrations, induces *Trypanosoma cruzi* cell death by necrosis in mitochondrion-dependent manner.
